# CRISPRi-mediated metabolic engineering of *E. coli* for *O*-methylated anthocyanin production

**DOI:** 10.1186/s12934-016-0623-3

**Published:** 2017-01-17

**Authors:** Brady F. Cress, Quentin D. Leitz, Daniel C. Kim, Teresita D. Amore, Jon Y. Suzuki, Robert J. Linhardt, Mattheos A. G. Koffas

**Affiliations:** 1 Department of Chemical and Biological Engineering, Center for Biotechnology and Interdisciplinary Studies, Rensselaer Polytechnic Institute, Biotech 4005D, 110 8th Street, Troy, NY 12180 USA; 2 Department of Tropical Plant and Soil Sciences, University of Hawaii, 3190 Maile Way, Honolulu, HI 96822 USA; 3 U.S. Department of Agriculture, Agricultural Research Service, Daniel K. Inouye U.S. Pacific Basin Agricultural Research Center, Hilo, HI 96720 USA; 4 Department of Biological Sciences, Center for Biotechnology and Interdisciplinary Studies, Rensselaer Polytechnic Institute, Troy, NY 12180 USA; 5 Department of Chemistry and Chemical Biology, Center for Biotechnology and Interdisciplinary Studies, Rensselaer Polytechnic Institute, Troy, NY 12180 USA

**Keywords:** CRISPRi, dCas9, Transcriptional repression, Deregulation, MetJ, Peonidin 3-*O*-glucoside, Anthocyanin *O*-methyltransferase, *S*-Adenosyl methionine, SAM, AdoMet

## Abstract

**Background:**

Anthocyanins are a class of brightly colored, glycosylated flavonoid pigments that imbue their flower and fruit host tissues with hues of predominantly red, orange, purple, and blue. Although all anthocyanins exhibit pH-responsive photochemical changes, distinct structural decorations on the core anthocyanin skeleton also cause dramatic color shifts, in addition to improved stabilities and unique pharmacological properties. In this work, we report for the first time the extension of the reconstituted plant anthocyanin pathway from (+)-catechin to *O*-methylated anthocyanins in a microbial production system, an effort which requires simultaneous co-option of the endogenous metabolites UDP-glucose and *S*-adenosyl-l-methionine (SAM or AdoMet).

**Results:**

Anthocyanin *O*-methyltransferase (AOMT) orthologs from various plant sources were co-expressed in *Escherichia coli* with *Petunia hybrida* anthocyanidin synthase (*Ph*ANS) and *Arabidopsis thaliana* anthocyanidin 3-*O*-glucosyltransferase (*At*3GT). *Vitis vinifera* AOMT (*Vv*AOMT1) and fragrant cyclamen ‘Kaori-no-mai’ AOMT (*Ckm*OMT2) were found to be the most effective AOMTs for production of the 3′-*O*-methylated product peonidin 3-*O*-glucoside (P3G), attaining the highest titers at 2.4 and 2.7 mg/L, respectively. Following modulation of plasmid copy number and optimization of *Vv*AOMT1 and *Ckm*OMT2 expression conditions, production was further improved to 23 mg/L using *Vv*AOMT1. Finally, CRISPRi was utilized to silence the transcriptional repressor MetJ in order to deregulate the methionine biosynthetic pathway and improve SAM availability for *O*-methylation of cyanidin 3-*O*-glucoside (C3G), the biosynthetic precursor to P3G. MetJ repression led to a final titer of 51 mg/L (56 mg/L upon scale-up to shake flask), representing a twofold improvement over the non-targeting CRISPRi control strain and 21-fold improvement overall.

**Conclusions:**

An *E. coli* strain was engineered for production of the specialty anthocyanin P3G using the abundant and comparatively inexpensive flavonol precursor, (+)-catechin. Furthermore, dCas9-mediated transcriptional repression of *metJ* alleviated a limiting SAM pool size, enhancing titers of the methylated anthocyanin product. While microbial production of P3G and other *O*-methylated anthocyanin pigments will likely be valuable to the food industry as natural food and beverage colorants, we expect that the strain constructed here will also prove useful to the ornamental plant industry as a platform for evaluating putative anthocyanin *O*-methyltransferases in pursuit of bespoke flower pigment compositions.

**Electronic supplementary material:**

The online version of this article (doi:10.1186/s12934-016-0623-3) contains supplementary material, which is available to authorized users.

## Background

Many plant natural products and polyphenols have been associated with a diverse array of health benefits, particularly in studies performed in vitro, but controversy surrounds many of these compounds with respect to their efficacy in humans despite the higher success rate of natural products versus other chemicals in Phase I testing [1]. Nonetheless, industrial interest in these products has stagnated because of difficulty sourcing large quantities of complex natural products, primarily due to low abundance of these compounds in raw plant extracts. Complicated syntheses suffer from low yield due to the presence of multiple stereocenters, and, thus, offer little hope as a viable alternative to extraction [2]. Despite these difficulties, the brightly colored plant pigments known as anthocyanins are intriguing candidates for continued investigation due to their antioxidant properties, reported health benefits, and high potential for use as natural food and beverage colorants [3] since they already pervade nearly all diets.

Anthocyanins are pigmented flavonoids that are classically considered to be naturally biosynthesized in plants through the subsequent conversion of flavanones to dihydroflavonols by flavanone 3β-hydroxylase (F3H), dihydroflavonols to leucoanthocyanidins by dihydroflavonol 4-reductase (DFR), and leucoanthocyanidins to anthocyanidins by the α-ketoglutarate- and Fe(II)-dependent anthocyanidin synthase (ANS). We have previously shown, however, that *Escherichia coli* expressing ANS are able to convert the flavan-3-ols afzelechin and catechin into the anthocyanidins pelargonidin and cyanidin, respectively [4]. This affords the opportunity for one-step biotransformation of the cheap and abundant precursor (+)-catechin into the unstable compound cyanidin, which can be significantly stabilized by glycosylation with UDP-glucose: 3-*O*-glycosyltransferase (3GT) to form cyanidin 3-*O*-glucoside. Such a strategy enables extension and exploration of the heterologous microbial anthocyanin biosynthetic pathway in the genetically tractable host, *E. coli*.

Traditional microbial workhorse hosts like *E. coli* and *Saccharomyces cerevisiae* remain an attractive option for both exploration of natural enzymatic tailoring reactions and production of specialty natural products requiring several biosynthetic steps, primarily due to their well-characterized metabolisms and the wealth of advanced genetic tools [1, 5, 6]. Additionally, flavonoids and anthocyanins are convenient molecules for probing the capacity for co-option of specific endogenous microbial cosubstrates and cofactors; these polyphenols are easy to quantify, and most are readily permeable to cell membranes through passive and active diffusion (an important feature for substrate feeding, mutasynthesis [7–10], and co-culture studies [11] requiring inter-strain transport of pathway intermediates). Synthetic biology platforms developed for combinatorial heterologous gene expression in these hosts further facilitate rapid examination of genes from disparate sources [12], enabling augmented synthetic pathway flux due to either improved enzymatic properties (*K*_*m*_ and *k*_*cat*_), reduced inhibition from pathway intermediates and end products, or distinct heterologous expression capacities exhibited by divergent gene orthologs.

Prior to this work, our lab has successfully reconstituted many short flavonoid [11, 13, 14] and anthocyanin [3, 4, 15, 16] pathway segments in *E. coli*, which served as important testbeds to study endogenous cosubstrate or cofactor limitations [17, 18] toward the ultimate objective of de novo microbial production of complex polyphenols (starting with glucose and requiring construction of long pathways of 10–20 heterologous biosynthetic genes). Until now, however, we have not significantly extended these preliminary studies toward exploration of anthocyanin decoration. Instead, we have focused primarily on UDP-glucose improvement—by supplementation or overexpression of relevant genes in central carbon metabolism—for conversion of the anthocyanidins (aglycones) pelargonidin and cyanidin into the anthocyanins (glycosides) pelargonidin 3-*O*-glucoside and cyanidin 3-*O*-glucoside [3, 4, 15]. Other common anthocyanin modifications of interest besides hydroxylation include *O*-methylation [19] and *C*-methylation [20], aliphatic acylation (acetylation and malonylation) or aromatic acylation (cinnamoylation, hydroxycinnamoylation, hydroxybenzoylation, etc.) [21], and various glycosylations with distinct hexose or pentose donors at different carbon positions on the core anthocyanin (including diglycosides or linear disaccharide/trisaccharide extensions at a single carbon on the core structure) [22–24]. These decorations are desirable because they increase product stability and thus facilitate characterization and quantification in complex matrices like cell cultures. Additionally, such modifications change photochemical properties, making these tailored products attractive as natural pigments for the food, beverage, and cosmetics colorant industries. A platform strain enabling rapid screening of putative anthocyanin tailoring enzymes from various plant sources using (+)-catechin or other inexpensive and abundant substrates would also prove useful for plant scientists.

Microbial anthocyanin *O*-methylation is one straightforward target modification that has not been examined to date in the context of a heterologous biosynthetic pathway. Instead, most anthocyanin *O*-methyltransferases (AOMTs) have been characterized *in planta* or in vitro, following purification of heterologously-expressed enzyme from *E. coli*. Given the existence of *S*-adenosylmethionine (SAM or AdoMet, the cosubstrate used by AOMTs to methylate anthocyanins primarily at the 3′- and 5′-hydroxyls) in the natural metabolism of *E. coli*, coupled with the proven capability of *E. coli* to express active AOMTs, we posited that endogenous SAM pools might be sufficient to achieve in vivo anthocyanin *O*-methylation. Further supporting this hypothesis, *E. coli* has been utilized as a host for heterologous production of other natural compounds that require SAM as a cosubstrate such as vanillate [25], *N*-acyl-homoserine lactones [26], polyketides [27], and even for biotransformations of flavonoids to their *O*-methylated counterparts [28–30]. Until recently, however, there has been little effort aimed at improving SAM pools for co-option by exogenous, SAM-dependent methyltransferase reactions. Primary strategies have been more traditional, including supplementation of l-methionine [31] (the immediate and limiting SAM precursor) and overexpression of SAM synthase (*metK*) [27–29], but recent advances in the field of synthetic biology are expediting our ability to explore novel metabolic engineering strategies. In particular, the advent of dCas9-mediated transcriptional repression (also known as CRISPR interference, or CRISPRi) has enabled rapid assessment of metabolic engineering interventions at a rate that was previously inaccessible [32, 33], predominantly due to the ease and specificity of targeting the programmable synthetic transcription factor dCas9 with a guide RNA (gRNA) designed to be complementary to a ~20 bp target DNA site [34]. Therefore, we turned to CRISPRi as a tool to quickly probe metabolic perturbations in *E. coli* for improved SAM availability and, as a result, increased anthocyanin *O*-methylation.

**Fig. 1:**
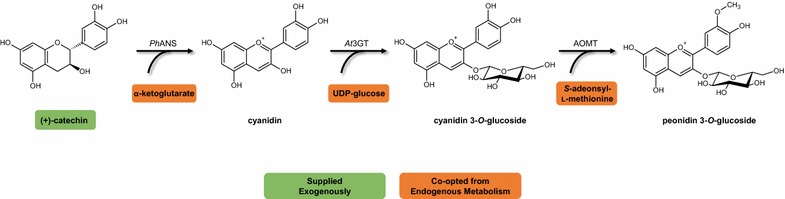
Heterologous anthocyanin pathway for conversion of the flavan-3-ol substrate (+)-catechin to the *O*-methylated anthocyanin product peonidin 3-*O*-glucoside (P3G). Substrate (*green*) was supplied in media, while cofactors (*orange*) required for each enzymatic step were co-opted from endogenous *E. coli* metabolism

In this work, we present three major findings. We successfully extend the microbial heterologous anthocyanin pathway to methylated anthocyanins for the first time, producing peonidin 3-*O*-glucoside (P3G) from the flavan-3-ol substrate (+)-catechin (Fig. 1). We demonstrate a simple alternative strategy for improving SAM availability for anthocyanin methylation by deregulating the methionine and SAM biosynthetic pathway, an approach that should be applicable for production of any methylated natural product. Finally, and in agreement with our previous work, we demonstrate that CRISPRi-mediated gene silencing is an effective tool for rapid assessment of metabolic engineering interventions, and we show that repression of transcriptional regulators can be a valuable tool for coordinated global metabolic perturbations.

## Results

### Anthocyanin *O*-methyltransferases from different organisms exhibit distinct in vivo production capacities

A common starting point for improving production of heterologous natural products in microbial hosts is screening of known and putative enzymes catalyzing the same reaction but isolated from different donor organisms [12]. Although the outcomes of such screens do not necessarily shed light on the rationale for exceptional performance of a particular ortholog in the context of the reconstituted pathway, these screens often uncover a gene or combination of genes that perform significantly better than others in a specific expression context. Further investigation is often required to determine if an ortholog has better inherent enzymatic properties, is less sensitive to feedback inhibition, or is simply expressed at a more optimal level for production (not necessarily higher, but perhaps more balanced with the other heterologous pathways). With this in mind, we selected five previously validated anthocyanin *O*-methyltransferases (AOMTs) from the literature to test for extension of our synthetic heterologous pathway. AnthOMT (referred to in this work as *Sl*AOMT for convenience) was originally characterized due to its role in anthocyanin diversification in tomato (*Solanum lycopersicum*) seedlings [35]. *Ckm*OMT2 was isolated as the major paralog—out of four in the genome of the fragrant cyclamen cultivar ‘Kaori-no-mai’—responsible for methylation of the anthocyanin delphinidin 3,5-diglucoside [36]. *Vv*AOMT1 from grape has been characterized as a flavonol and anthocyanin 3′, 5′ *O*-methyltransferase [37–39]. *Ph*MF1 and *Ph*MF2 are closely related paralogs from *Petunia hybrida* [37].

**Fig. 2:**
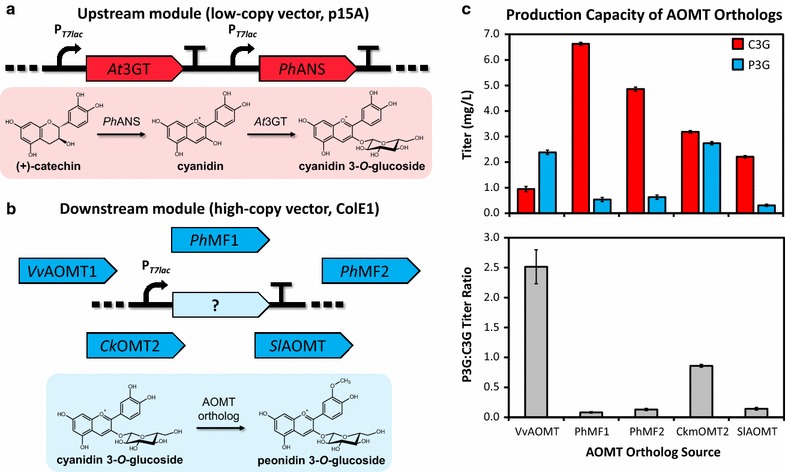
Modular ortholog screen identifies several AOMTs that are capable of producing peonidin 3-*O*-glucoside (P3G) in *E. coli*. **a** The upstream module, encoded on low-copy ePathBrick vector pACM4, produces cyanidin 3-*O*-glucoside (C3G) from (+)-catechin and endogenous UDP-glucose, **b** all AOMT orthologs were cloned into compatible high-copy ePathBrick vector pETM6 to facilitate rapid screening of downstream modules following combinatorial co-transformation with upstream module. The downstream module converts C3G and endogenous *S*-adenosylmethionine (SAM) to P3G, **c** ortholog screen reveals *Vv*AOMT1 and *Ckm*OMT2 as best P3G (*blue*) producers overall (*top*), while *Vv*AOMT1 achieved best conversion of the final C3G to P3G step as demonstrated by the high final P3G:C3G titer ratio (*bottom*)

After all AOMTs were cloned into a high-copy vector (~50 copies) and sequence verified, each individual ortholog was co-expressed in modular fashion with a *Ph*ANS and *At*3GT cassette harbored on a compatible but lower copy vector (~15 copies) (Fig. 2a, b). Using this dual plasmid system, no additional cloning was required to compare orthologs. As seen in Fig. 2c, all AOMTs that were screened here were capable of performing *O*-methylation of C3G to P3G in *E. coli*, marking what we believe to be the first example of engineered, multi-step microbial anthocyanin methylation reported to date. A strain lacking AOMT did not generate P3G, indicating that *E. coli* BL21 lacks endogenous anthocyanin *O*-methylation capability. Of note, AOMT orthologs from grape (*Vv*AOMT1) and cyclamen (*Ckm*OMT2) showed significant improvement in production capacity over the other examined orthologs, although even these two genes yielded relatively low titers (Fig. 2c, top). The relative production capacity differences between the orthologs may be due in part to substrate preferences; for example, data suggest that grape AOMT methylates anthocyanin 3-glycoside derivatives more efficiently than petunia AOMT orthologs that exhibit higher activity for anthocyanin (3-acyl) rutinoside 5-glucoside substrates [37, 40]. Coupled with potential differences in AOMT ortholog expression efficiencies, these differences in substrate affinity could impact the conversion rate from C3G to P3G and lead to the disparate overall titers (C3G plus P3G) observed between strains. Despite achieving similar P3G titers, *Vv*AOMT1 demonstrated nearly threefold higher conversion from C3G to P3G than *Ckm*OMT2 (Fig. 2c, bottom) in this pathway configuration.

### Gene dosage affects C3G and P3G titers

Given the low overall titers of both C3G and P3G obtained during the homolog screen, we hypothesized that substrate utilization and, thus, overall production would be improved by increasing expression of the upstream genes. We next evaluated the effect of gene dosage (mediated by plasmid copy number modulation) on P3G production. Expression of the upstream module composed of *Ph*ANS and *At*3GT was increased by transferring from the low-copy vector into high-copy vectors harboring one of the two best AOMTs (*Vv*AOMT1 and *Ckm*OMT2). In addition, an alternative upstream module composed of maltose binding protein (MBP)-tagged *Ph*ANS and *At*3GT was tested with each of the two best AOMTs in parallel. Although no strict hypothesis was posed as rationale for improved production using MBP tags, we had previously achieved high concentrations of soluble MBP-*Ph*ANS and MBP-*At*3GT during in vitro characterization (unpublished data), perhaps because cytosolic MBP (N-terminal membrane domain removed) is known to enhance solubility of its fusion partners [41]. Moreover, modulation of protein translation rate through alteration of ribosome binding site (RBS) and N-terminal sequence (by fusion tag or alternative codon usage) is one of many methods used to balance expression of genes within a pathway [42, 43]. We thus speculated that even slight variation of upstream pathway expression due to the presence of N-terminal MBP tags could cause significant changes in production either through improved solubility of the upstream enzymes or through the resulting altered balance with the downstream AOMT.

**Fig. 3:**
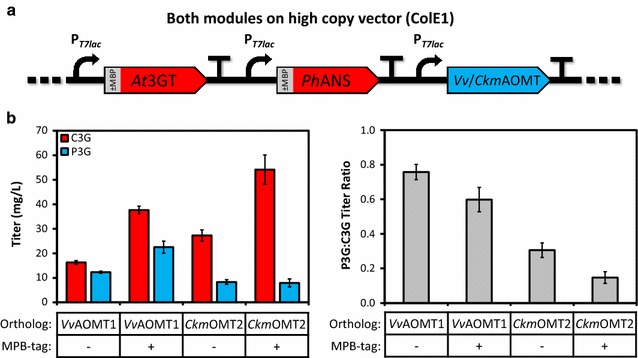
Gene dosage affects production of P3G. **a** Specifically, the upstream module was transferred from a low-copy vector into a single cassette with *Vv*AOMT1 or *Ckm*OMT2 on a high-copy vector, **b** increased copies of the upstream module led to ~fivefold improvement in P3G titer over the original constructs (Fig. 2). An additional, modest twofold improvement in overall P3G production (*left*), accompanied by a slight decrease in C3G to P3G conversion (*right*), was further achieved with *Vv*AOMT1 (*left*) by utilizing an upstream module composed of MBP-fusions

In total, four high-copy pathway variants were examined as shown in Fig. 3a. Shifting the upstream module from a low to high-copy plasmid dramatically improved accumulation of both C3G and P3G. However, conversion from C3G to P3G was dampened relative to the initial screen, potentially highlighting an imbalance between the upstream and downstream modules. Compared to production levels achieved with the upstream module at low copy number, a higher gene dosage of *Ph*ANS and *At*3GT improved production by ~fivefold and is clearly important for driving flux into the heterologous pathway. Interestingly, however, utilization of MBP tags on the upstream enzymes improved production only when coupled with *Vv*AOMT1, while not affecting final titer when coupled with *Ckm*OMT2. It is not clear if the MBP tag improved production in combination with *Vv*AOMT1 due to altered solubility and thus modulated intra-pathway enzyme balance, due to altered translation rate, or due to some other unknown factor. In the end, the best high-copy configuration, which is composed of the MBP-tagged upstream module and *Vv*AOMT1, exhibited ~ninefold higher production than the best configuration from the ortholog screen. Despite the significantly improved production of P3G, high levels of remaining intermediate C3G suggested a potential limitation in cofactor availability for *O*-methylation of C3G. It should be noted here that increased gene dosage does not always correlate with increased protein production or increased metabolic flux; as such, we consider gene dosage modulation (through copy number alteration or transcriptional/translational control) as another strategy to alter expression context, which can sometimes lead to improved production as seen in this work [44].

### CRISPRi-mediated deregulation of methionine and *S*-adenosyl methionine biosynthesis improves P3G titers

While we have previously shown that endogenous pools of UDP-glucose are limiting for production of the anthocyanins C3G and pelargonidin 3-*O*-glucoside (similar to C3G, but monohydroxylated at the para-position of the B ring) from their corresponding aglycones in *E. coli* [4], we sought in this work to explore the potential limitation of SAM availability for *O*-methylation of anthocyanins. Several approaches have been previously utilized to overproduce SAM in *E. coli*, but one surprisingly neglected strategy posed at the initiation of this work was deregulation of SAM and methionine biosynthesis by simply silencing *metJ*, the ligand-responsive transcriptional repressor that naturally regulates production of methionine and SAM in response to feedback from SAM accumulation (and, to a lesser degree, accumulation of other structurally similar ligands) [45]. Although pathway deregulation is not always sufficient to engender accumulation of biosynthetic intermediates, it is often successful because of the coordinated response—in some cases simultaneous repression, de-repression, and activation of genes at key regulatory nodes—that has naturally evolved to drive flux through a pathway in the absence of a feedback regulator’s cognate ligand (or in the presence of the cognate ligand, depending on whether a transcription factor binds or releases DNA in response to association with the ligand).

**Fig. 4:**
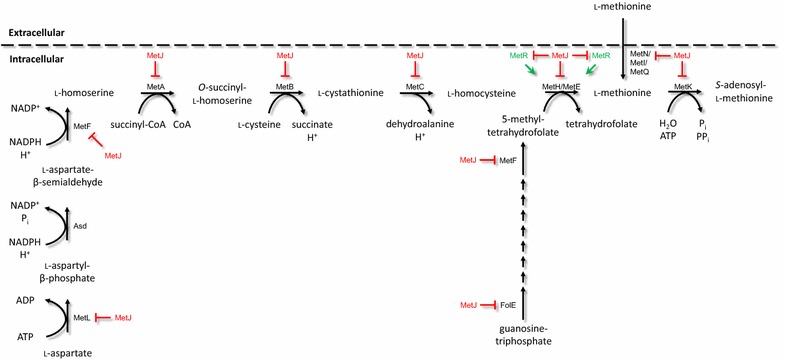
Superpathway of methionine and SAM biosynthesis, with known transcriptional regulatory constraints indicated. Many genes involved in methionine biosynthesis and active, ATP-dependent uptake are part of the MetJ regulon and are thus subject to strong negative regulation (repression) by the eponymous ligand-responsive transcription factor MetJ (*red*) in presence of its cognate ligand SAM. Furthermore, the endogenous transcriptional activator of methionine synthase known as MetR (*green*) is repressed by MetJ:SAM, further attenuating methionine and SAM accumulation through de-activation of a critical node in the methionine biosynthetic pathway

In prior work, we demonstrated the power of pathway deregulation to improve production of a target metabolite with a single, focused genetic intervention intended to globally coordinate metabolism toward a defined objective. Specifically, we previously utilized CRISPRi/dCas9-mediated transcriptional repression of *fadR*, the dual regulator of fatty acid biosynthesis and degradation, to enhance naringenin production through increased intracellular malonyl-CoA pool [32]. In the current effort, we implemented a similar approach to deregulate the methionine and SAM biosynthetic pathways using a single, pointed intervention intended to coerce *E. coli* to drive metabolic flux toward SAM through the otherwise highly regulated methionine pathway including the critical methionine synthase (MetH/MetE) node. In the presence of high SAM concentrations, the ligand-responsive transcriptional regulator MetJ is known to repress at least 12 genes from 9 promoters related to methionine and SAM biosynthesis in *E. coli* (Fig. 4) [46]. We hypothesized that de-repression of the complete methionine and SAM biosynthetic pathways, mediated by the silencing of *metJ*, would result in increased SAM pools and thereby increased *O*-methylation of C3G.

**Fig. 5:**
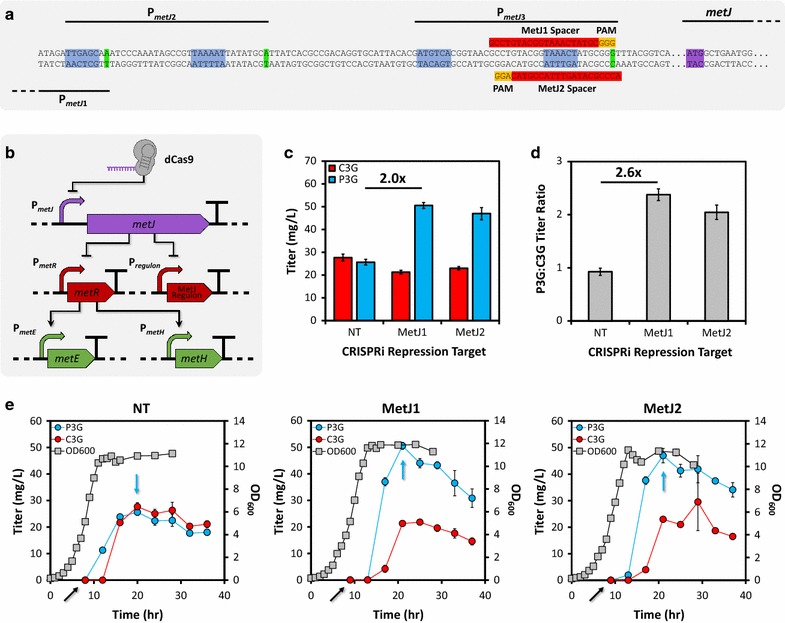
CRISPRi-mediated improvement of P3G production through increased SAM availability. **a** MetJ promoter (P_*metJ*_) region architecture with anti-*metJ* CRISPR elements indicated (spacers in *red*, PAMs in *yellow*). Three promoters drive *metJ* expression (+1 sites in *green* and −35 and −10 sites for P_*metJ*2_ and P_*metJ*3_ in *blue*), so CRISPR spacers were designed to target the downstream promoter P_*metJ*3_ in order to simultaneously repress transcription from all three promoters, collectively indicated as P_*metJ*_, **b** schematic representation of the regulatory cascade engendered by CRISPRi-mediated deregulation of the *metJ* regulon. Repression of MetJ de-represses the *metJ* regulon, which is composed of at least twelve genes and nine promoters involved in methionine and SAM biosynthesis, including MetR (*red*). De-repression of MetR prompts activation of methionine synthase (*metH*/*metE* in *green*), the final step in methionine biosynthesis immediately preceding SAM synthase. The overall effect of MetJ silencing is unregulated, increased flux through the methionine and SAM biosynthetic pathways, **c** both anti-*metJ* CRISPR spacers achieved twofold increase in P3G titer and approximately 2.5-fold increase in C3G to P3G conversion (**d**) relative to the non-targeting, scrambled CRISPR negative control spacer (NT), **e** sampling time course demonstrates that C3G (*red circles*) and P3G (*blue circles*) degradation occurs in culture and that optimization is required to reduce the detrimental effect of degradation on final titers, **c**, **d** utilize maximum titers obtained from optimal sampling conditions (*blue arrows*). Induction of slightly slower growing MetJ-repression strains (*middle* and *right*) was delayed by 1 h (*black arrows*) to ensure OD_600_ (*gray squares*) at induction was identical to NT strain (*left*), specifically corresponding to pre-determined optimal mid-log phase induction at OD_600_ ~5.1–5.4

Toward this goal, two CRISPR target sites (spacers) were selected 5′ to the edge of existing NGG trinucleotides known as protospacer-adjacent motifs (PAMs) [47] and overlapping the −10 element of promoter P_*metJ*3_, the third and furthest downstream of three MetJ promoters (P_*metJ*1_, P_*metJ*2_, and P_*metJ*3_). Targeting this location should cause dCas9-mediated occlusion of RNA polymerase from the third promoter (closest to the *metJ* start codon) and roadblocking of potential transcripts initiated from both upstream promoters (Fig. 5a). The outcome of *metJ* silencing is illustrated in Fig. 5b, which succinctly depicts the ensuing dysregulated signaling cascade. In the absence of MetJ, at least 12 genes required for production of SAM, through methionine biosynthesis and uptake, are de-repressed. Of note, the ligand-responsive transcriptional dual regulator MetR naturally activates transcription of methionine synthase (MetH/MetE) in presence of high concentrations of its cognate ligand l-homocysteine (immediate methionine precursor). Thus, the increased expression of MetR following its de-repression likely complements elevated levels of homocysteine, resulting in activation of methionine synthase; this can occur even at increased SAM concentrations due to the absence of MetJ, which would otherwise repress methionine synthase and upstream genes in response to elevated SAM.

To test the hypothesis that CRISPRi-mediated repression of *metJ* would improve SAM availability for *O*-methylation, compatible vectors bearing constitutive CRISPRi elements (dCas9, tracrRNA, and a minimal single-spacer CRISPR array) were co-transformed with the best vector from the gene dosage experiment (*Vv*AOMT1 with MBP-tagged upstream module on high-copy vector). Interestingly, C3G titer was affected by addition of the non-targeting CRISPRi control vector to this strain. Although it is not clear why this occurred, we speculate that previously reported growth inhibition associated with expression of dCas9 and crRNA/gRNA [48] slightly altered the production capacity or induction optimum for this strain. Regardless, compared to the non-targeting spacer (previously used as a negative control in *E. coli* by other groups [49] and ours [32, 50]), both anti-*metJ* spacers improved production of P3G by approximately twofold (Fig. 5c), representing approximately 20-fold improvement over the best strain from the initial screen. Critically, the conversion of C3G to P3G by AOMT was also markedly improved by MetJ repression (Fig. 5d), which would likely be expected if AOMT’s cosubstrate SAM were limiting. Additionally, it is recommended that multiple gRNAs (or spacers) be selected against each CRISPRi target due to our current inability accurately predict gRNA (spacer) efficiency and specificity [51]. In this work, testing more than one *metJ* dCas9-binding site demonstrated two important points. Targeting one site achieved slightly higher production than targeting the other site in a similar location, supporting the notion that non-intuitive changes in dCas9-binding site selection can affect production level. Secondly, similar improvements in C3G to P3G conversion for both *metJ* repression targets relative to the non-targeting control imply that improved production was indeed due to *metJ* repression; in other words, use of alternative dCas9-binding sites against a single target gene or promoter can serve as a specificity control for the assayed phenotype. Finally, the best *metJ* repression strain from this experiment (MetJ1) was used to demonstrate cultivation scale-up from 1 mL scale in multi-well plates to 25 mL working volume in 125 mL non-baffled shake flasks. Production improved slightly upon scale-up to a final titer of 56.3 ± 0.6 mg/L (mean ± SEM of three independent experiments), a 21-fold improvement over the best strain from the ortholog screen.

## Discussion

This report details microbial *O*-methylated anthocyanin production as a case study for anthocyanin decoration in a platform strain, and the results indicate that the endogenous *E. coli* SAM pool is sufficient for designer methylation reactions but can be improved by targeted genetic interventions. For example, although SAM availability in *E. coli* has been the focus of a few other recent engineering efforts for *O*-methylation of natural products, the methionine and SAM biosynthesis deregulation strategy described herein could also facilitate in vivo characterization of SAM-dependent methyltransferases catalyzing C-, N-, and S-dependent methylation of other natural products [52]. While our lab has strived in the past to systematically extend and connect flavonoid and anthocyanin pathways by improving cofactor and cosubstrate availability, this work was instead intended to demonstrate that underexplored anthocyanin tailoring reactions can be rapidly tested in a model microbial system using the inexpensive and abundant substrate (+)-catechin. We anticipate that additional enzymatic reactions will be tested by others using a similar strategy, but consideration should be devoted to availability of cognate cosubstrates; if a dearth of putative orthologs exists for a reaction of interest, it is conceivable that low intracellular cosubstrate concentration will impede conversion and product characterization. In any case, CRISPRi is a quick and easy alternative to gene deletion to improve metabolite pools. A significant limitation to this platform strain is that products are constrained to C3G (3′-, 4′-hydroxylated) as a precursor when feeding (+)-catechin as a substrate. There are a vast number of tailoring reactions that should be compatible with this C3G, but methylated anthocyanidins like petunidin and malvidin and their glycosides are inaccessible unless an appropriate upstream substrate can be synthesized (difficult to control stereochemistry) [53] or biosynthesized (not achieved to date, and P450 hydroxylation is quite difficult in *E. coli*).

In addition to screening and characterizing anthocyanin tailoring reactions, the platform described here could ultimately prove feasible for industrial production of modified anthocyanins. Despite modest titers outlined here for the novel anthocyanin product P3G, very impressive and extensive work published during the preparation of this manuscript examined several solutions to SAM limitation in *E. coli* K-12 MG1655(DE3) lineages for production of vanillate, an *O*-methylated natural product that had been produced in *E. coli* previously but suffered from low titers [25]. Combined deregulation of SAM biosynthesis through *metJ* deletion, feedback desensitization of enzymes catalyzing committed or important biosynthetic steps, and deregulation of SAM regeneration improved de novo vanillate titers by twofold to approximately 400 mg/L [25]. These findings imply that SAM-dependent natural product biosyntheses in *E. coli* at industrially relevant titers will likely be achievable. Interestingly, and in contrast to our work with anthocyanins, deletion of *metJ* alone was not sufficient to improve vanillate titers. It is possible that this difference stems from disparities in metabolite pools or regulation between B (used in this work) and K (used in [25]) *E. coli* lineages; alternatively, this could be attributed simply to distinct enzyme properties between the selected AOMT and catechol OMT used for vanillate production. Other strategies outlined in literature that have been reported to augment SAM production include supplementation of media with methionine and improvement of precursor ATP pools by reducing ATP usage in other reactions [54]. Another tactic that has not been tested but would likely prove synergistic with MetJ deletion in conjunction with these other strategies is overexpression of MetR, as it has been shown that the combination of SAM:MetJ shortage with overproduction of MetR induces overexpression of methionine synthase [55]. Nonetheless, a combination of all the aforementioned interventions with the UDP-glucose improvements previously described by our lab could eventually lead to industrially relevant titers for these high-value, complex specialty anthocyanins.

Finally, although there are limitations to using CRISPRi rather than gene deletions (potential for incomplete repression, commonly constructed on plasmid-based systems requiring antibiotics and prone to instability, etc.), there are significant advantages that make CRISPRi the unsurpassed tool for targeted gene silencing that exists today. First, design of CRISPRi constructs is swift and straightforward. CRISPRi vectors can be cloned, transformed into production strains, and assessed with predictable results 2–3 days after target selection. If designed appropriately, inducible CRISPRi also enables complete repression (silencing) or tunable, partial repression of essential genes at any point of the growth phase, such as when sufficient biomass has accumulated; a related, highly attractive feature is the capacity to partially repress and somewhat predictably tune both essential and nonessential genes with CRISPRi. Multiplexing can even be achieved following rapid hierarchical assembly of gRNAs or CRISPR arrays, and spacers that have been designed against conserved sequences in multiple strains are portable—i.e. the vectors can be transformed into any host background, facilitating faster assessment of multiple downregulations in disparate strains than any other existing method. Finally, CRISPRi can be used to gently coax cells into a rewired state with little effort; specifically, *metJ* repression is a “soft” strategy that achieves widespread metabolic perturbation (de-repression of many genes in the same pathway, simultaneously) without the metabolic burden associated with high-copy overexpression of many heterologous genes at once [56]. We anticipate that similar CRISPRi-mediated, systems-level interventions will expedite understanding of how new cosubstrates can be co-opted for novel anthocyanin decorations.

## Conclusions

In this study, we have engineered a strain to serve as a platform for modular, rapid examination of anthocyanin tailoring enzymes. The strain only requires supplementation of the inexpensive and abundant precursor (+)-catechin to generate anthocyanins for downstream modification. We exemplify the utility of this strain by engineering microbial production of an *O*-methylated anthocyanin for the first time. Combining classical metabolic engineering principles and cutting-edge synthetic biology approaches, we significantly improved production of our target compound P3G and gained insight about co-opting limiting AOMT cosubstrate pools. First, we screened AOMTs from disparate sources to isolate candidates with improved in vivo production capacities, a tried-and-true approach that should be considered at the outset of any metabolic engineering project. Next, we transferred the upstream genes to a high copy plasmid. The result was significantly improved flux, suggesting that these genes benefit from increased copy but implying that the effect of gene dosage on product titer should be assayed to explore expression space. Finally, we found that coordinated, global perturbation of SAM biosynthesis was obtainable through a single CRISPRi intervention, leading to increased conversion through the anthocyanin *O*-methylation tailoring reaction. The *metJ* dysregulation strategy described in this and another recent report [25] should be useful for improving methylation of any natural product, but the general steps taken here should also be applicable for studying unique anthocyanin tailoring reactions that require co-option of distinct cosubstrates. Future efforts to integrate anthocyanin pathway genes into the genome of *E. coli* should also be undertaken to generate stable production strains lacking plasmids.

## Methods

### Strains and plasmids

A list of all *E. coli* strains and plasmids constructed in this work is provided in Table 1. All ortholog plasmids, pETM6 production plasmids, and CRISPRi plasmids generated for this manuscript have been made available to the community through Addgene (plasmids #86901-86917). Plasmid construction and propagation was performed in *E. coli* DH5α™. All anthocyanin production experiments were performed using *E. coli* BL21 Star™ (DE3) as a host for anthocyanin pathway and CRISPRi plasmids. Upstream genes *Ph*ANS and *At*3GT were obtained by PCR amplification (ACCUZYME mix, Bioline) using plasmid pMAL-c2X-*Ph*ANS (unpublished) as a template, followed by traditional restriction ligation cloning into the ePathBrick destination vector pETM6. Fusions of *Ph*ANS and *At*3GT with cytosolic MBP (truncated in pMAL-c2X to remove the N-terminal MBP membrane domain) were obtained in a similar manner, but the forward primer was designed to bind near the MBP tag start codon on pMAL-c2X in order to amplify the full fusion coding sequence through the stop codon of *Ph*ANS or *At*3GT. The five anthocyanin *O*-methyltransferase orthologs were synthesized as gBlocks (Integrated DNA Technologies) maintaining wild-type sequences, with any problematic internal restriction sites removed by silent mutation. Generic flanking regions were added to each gBlock, and universal primers M13F universal and M13R universal were used to amplify each AOMT prior to cloning into destination vector pETM6. The sequences of all primers used in this study are listed in Table 2. All gBlock sequences are provided in Additional file 1: Table S1.

**Table 1:** Strains and plasmids used in this study

Strain or plasmid name	Properties/genotype	References
*E. coli* strains		
*E. coli* DH5α™	F-Φ80*lac*ZΔM15 Δ(*lac*ZYA-*arg*F) U169 *rec*A1 *end*A1 *hsd*R17 (rK−, mK+) *pho*A *sup*E44 λ− *thi*-1 *gyr*A96 *rel*A1	Novagen
*E. coli* BL21 Star™ (DE3)	F-*ompT hsdS*_B_ (rB^−^mB^−^) *gal dcm rne131* (DE3)	Invitrogen
*E. coli* plasmids		
pMAL-c2X-*Ph*ANS	ColE1(Amp^R^), *Petunia hybrida* anthocyanidin synthase (*Ph*ANS) with N-terminal cytosolic MBP tag	Unpublished
pMAL-c2X-*At*3GT	ColE1(Amp^R^), *Arabidopsis thaliana* 3-*O*-glucosyltransferase (*At*3GT) with N-terminal cytosolic MBP tag	Unpublished
pETM6-*Ph*ANS	ColE1(Amp^R^), ePathBrick feature, *Ph*ANS	This study
pETM6-*At*3GT	ColE1(Amp^R^), ePathBrick feature, *At*3GT	This study
pETM6-MBP-*Ph*ANS	ColE1(Amp^R^), ePathBrick feature, *Ph*ANS with N-terminal MBP tag	This study
pETM6-MBP-*At*3GT	ColE1(Amp^R^), ePathBrick feature, *At*3GT with N-terminal MBP tag	This study
pETM6-*At*3GT-m-*Ph*ANS	ColE1(Amp^R^), ePathBrick feature, *Ph*ANS and *At*3GT in monocistronic configuration	This study
pACM4-*At*3GT-m-*Ph*ANS	pACYC184(CmR), ePathBrick feature, *Ph*ANS and *At*3GT in monocistronic configuration	This study
pETM6-MBP-*At*3GT-m-MBP-*Ph*ANS	ColE1(Amp^R^), ePathBrick feature, *Ph*ANS and *At*3GT with N-terminal MBP tags in monocistronic configuration	This study
pETM6-*Vv*AOMT1	ColE1(Amp^R^), ePathBrick feature, *Vitis vinifera* anthocyanin *O*-methyltransferase (*Vv*AOMT1)	This study
pETM6-*Ckm*OMT2	ColE1(Amp^R^), ePathBrick feature, fragrant cyclamen ‘Kaori-no-mai’ anthocyanin *O*-methyltransferase 2 (*Ckm*OMT2)	This study
pETM6-*Ph*MF1	ColE1(Amp^R^), ePathBrick feature, *Petunia hybrida* anthocyanin *O*-methyltransferase 1 (*Ph*MF1)	This study
pETM6-*Ph*MF2	ColE1(Amp^R^), ePathBrick feature, *Petunia hybrida* anthocyanin *O*-methyltransferase 2 (*Ph*MF2)	This study
pETM6-*Sl*AOMT	ColE1(Amp^R^), ePathBrick feature, *Solanum lycopersicum* anthocyanin *O*-methyltransferase (*Sl*AOMT)	This study
pETM6-*Vv*AOMT1-m-*At*3GT-m-*Ph*ANS	ColE1(Amp^R^), ePathBrick feature, *Vv*AOMT1, *Ph*ANS, and At3GT in monocistronic configuration	This study
pETM6-*Vv*AOMT1-m-MBP-*At*3GT-m-MBP-*Ph*ANS	ColE1(Amp^R^), ePathBrick feature, *Vv*AOMT1 and N-terminal MBP-tagged *Ph*ANS and *At*3GT in monocistronic configuration	This study
pETM6-*Ckm*OMT2-m-*At*3GT-m-*Ph*ANS	ColE1(Amp^R^), ePathBrick feature, *Ckm*OMT2, *Ph*ANS, and At3GT in monocistronic configuration	This study
pETM6-*Ckm*OMT2-m-MBP-*At*3GT-m-MBP-*Ph*ANS	ColE1(Amp^R^), ePathBrick feature, *Ckm*OMT2 and N-terminal MBP-tagged *Ph*ANS and *At*3GT in monocistronic configuration	This study
pdCas9	pACYC184(Cm^R^), tracrRNA, *cas9*(D10A, H840A), non-targeting CRISPR spacer with two BsaI sites	[49]
pdCas9-M-MetJ1	pACYC184(Cm^R^), tracrRNA, *cas9*(D10A, H840A), CRISPR spacer targeting MetJ promoter at position 1	This study
pdCas9-M-MetJ2	pACYC184(Cm^R^), tracrRNA, *cas9*(D10A, H840A), CRISPR spacer targeting MetJ promoter at position 2	This study

**Table 2:** Primers and oligonucleotides used in this study

Name	Nucleotide sequence (5′ → 3′)	Cognate crRNA [Proto]spacer sequence (5′ → 3′)	Cognate PAM (5′ → 3′)
M13F universal	GTTTTCCCAGTCACGACGTTG	N/A	N/A
M13R universal	TGAGCGGATAACAATTTCACACAG	N/A	N/A
ANS_XbaI_F	CCCTCTAGAAATAATTTTGTTTAACTTTAAGAAGGAGATATACATATGGTGAATGCAGTAGTTAC	N/A	N/A
ANS_XhoI_R	CGATCTCGAGCTATTTAGATTCTTCAGCAGCAAC	N/A	N/A
3GT_NdeI_F	GCATCATATGACCAAACCCTCCGACC	N/A	N/A
3GT_XhoI_R	CGATCTCGAGTCAAATAATGTTTACAACTGCATCC	N/A	N/A
MBP_soluble_XbaI_F	CCCTCTAGAAATAATTTTGTTTAACTTTAAGAAGGAGATATACATATGAAAATCGAAGAAGGTAAACTGG	N/A	N/A
MetJ1_KD_F	AAACTCACGGTAACGCCTGTACGGTAAACTATGCG	GCCTGTACGGTAAACTATGC	GGG
MetJ1_KD_R	AAAACGCATAGTTTACCGTACAGGCGTTACCGTGA		
MetJ2_KD_F	AAACCTGACCGTAAACCCGCATAGTTTACCGTACG	ACCCGCATAGTTTACCGTAC	AGG
MetJ2_KD_R	AAAACGTACGGTAAACTATGCGGGTTTACGGTCAG		

Underline indicates full protospacer sequence

For cloning of individual genes, PCR amplicons and entry backbones were digested with the following restriction enzyme (FastDigest, Thermo Fisher Scientific) combinations: *Ph*ANS (XbaI/XhoI), *At*3GT (NdeI/XhoI), MBP-*Ph*ANS (XbaI/XhoI), MBP-*At*3GT (XbaI/XhoI), *Vv*AOMT1 (NdeI/XhoI), *Ckm*OMT2 (NdeI/XhoI), *Ph*MF1 (NdeI/XhoI), *Ph*MF2 (NdeI/XhoI), *Sl*AOMT (NdeI/XhoI), pETM6 (NdeI/XhoI or XbaI/XhoI). Digested amplicons and backbones were gel purified (E.Z.N.A MicroElute Gel Extraction Kit, Omega Bio-tek) and ligated (Rapid DNA Ligation Kit, Thermo Fisher Scientific) as appropriate to construct the single-gene vectors listed in Table 1. Ligation products were transformed into DH5α and confirmed by Sanger sequencing (GENEWIZ, Inc.).

Using the ePathBrick subcloning procedure [57], *At*3GT and *Ph*ANS were then assembled into monocistronic configuration (each gene flanked by its own T7 promoter and terminator) by ligation of restriction digestion fragments from plasmid pETM6-*At*3GT (NheI/SalI) and pETM6-*Ph*ANS (AvrII/SalI), yielding plasmid pETM6-*At*3GT-m-*Ph*ANS. MBP-*At*3GT and MBP-*Ph*ANS fusions were assembled into monocistronic configuration by ligation of restriction digestion fragments from plasmid pETM6-MBP-*At*3GT (NheI/SalI) and pETM6-*Ph*ANS (AvrII/SalI), yielding plasmid pETM6-MBP-*At*3GT-m-MBP-*Ph*ANS. For co-expression of pETM6-harbored AOMTs with *Ph*ANS and *At*3GT, the two upstream genes were excised as a single cassette from pETM6-*At*3GT-m-*Ph*ANS (AvrII/SalI) and subcloned into low-copy vector pACM4 (AvrII/SalI) to generate plasmid pACM4-*At*3GT-m-*Ph*ANS. Initial screening of AOMT orthologs was performed by co-electroporation of all five pETM6-based AOMTs with ePathBrick-compatible pACM4-*At*3GT-m-*Ph*ANS into BL21 Star™ (DE3).

Next, the two best AOMTs, *Vv*AOMT1 and *Ckm*OMT2, were combined with either PhANS-m-At3GT or MBP-PhANS-m-MBP-At3GT on the high-copy pETM6 backbone. Ligation of pETM6-*Vv*AOMT1 (NheI/SalI) with pETM6-*At*3GT-m-*Ph*ANS (AvrII/SalI) or pETM6-MBP-*At*3GT-m-MBP-*Ph*ANS (AvrII/SalI) yielded plasmids pETM6-*Vv*AOMT1-m-*At*3GT-m-*Ph*ANS and pETM6-*Vv*AOMT1-m-MBP-*At*3GT-m-MBP-*Ph*ANS, respectively. Similarly, ligation of pETM6-*Ckm*OMT2 (NheI/SalI) with pETM6-*At*3GT-m-*Ph*ANS (AvrII/SalI) or pETM6-MBP-*At*3GT-m-MBP-*Ph*ANS (AvrII/SalI) generated plasmids pETM6-*Ckm*OMT2-m-*At*3GT-m-*Ph*ANS and pETM6-*Ckm*OMT2-m-MBP-*At*3GT-m-MBP-*Ph*ANS, respectively.

Plasmids used for CRISPRi/dCas9-mediated transcriptional repression were assembled as described previously [32, 50]. All CRISPRi plasmids harbored dCas9, tracrRNA, and a minimal CRISPR array containing a single spacer. Each CRISPR element is transcriptionally controlled by native *Streptococcus pyogenes* constitutive promoters as in our previous work [32, 50]. Plasmid pdCas9, encoding a non-targeting spacer, was utilized as a negative control for CRISPRi interventions and as a base plasmid for cloning *metJ* repression spacers. For both *metJ* repression targets, two 35 bp complementary and slightly offset oligonucleotides (Integrated DNA Technologies) containing the spacer sequences for dCas9 targeting were phosphorylated with T4 polynucleotide kinase (PNK, New England Biolabs) and annealed (37 °C for 30 min, 98 °C for 5 min, ramp down to 25 °C over 15 min) to build inserts for each *metJ*-repressor variant. Complementary offset oligos possessed the following sequences: 5′-AAACN_30_G-3′ and 5′-N_30_CAAAA-3′, where N_30_ represents the genomic protospacer (target) sequence. Although spacer sequences in this CRISPR system are ~30 bp, it has previously been shown that ~10 bp are typically trimmed from the 5′ end of the spacer during crRNA-tracrRNA processing by RNAse III, yielding functional spacers of ~20 bp [47]. It is possible, however, that certain crRNAs retain the full length spacer as shown possible in the Type I-E CRISPR system from *E. coli* [58], but the effect that this would have on dCas9-mediated transcriptional repression of distinct targets in *E. coli* is currently unknown.

Phosphorylated and annealed inserts were then cloned into the recipient vector, pdCas9, at two adjacent BsaI sites in the minimal, single-spacer CRISPR array using a one-pot Golden Gate reaction with BsaI (New England Biolabs) and T7 DNA ligase (New England Biolabs). Plasmid pdCas9 was a gift from Luciano Marraffini (Addgene plasmid #46569). See Table 2 for a list of oligonucleotides used for construction of CRISPRi plasmids and their corresponding protospacer sequences.

### Culture conditions

To facilitate rapid screening of constructs and expression conditions, all experiments were performed in 1 mL cultures in polypropylene deep 48-well plates (5 mL, VWR) covered with breathable rayon film (VWR). A semi-rich defined media known as AMM, supplemented with 2% glucose and appropriate antibiotics (80 μg/mL ampicillin and 25 μg/mL chloramphenicol, as necessary), was utilized for all liquid culture growth, including overnights and production experiments. AMM has been described previously [32] and is composed of 3.5 g/L KH_2_PO_4_, 5.0 g/L K_2_HPO_4_, 3.5 g/L (NH_4_)_2_HPO_4_, 2 g/L casamino acids, 100 mL 10× MOPS mix (83.72 g/L MOPS, 7.17 g/L tricine, 28 mg/L FeSO_4_·7H_2_O, 29.2 g/L NaCl, 5.1 g/L NH_4_Cl, 1.1 g/L MgCl_2_, 0.48 g/L K_2_SO_4_, and 0.2 mL micronutrient stock), 1 mL 1 M MgSO_4_, 0.1 mL 1 M CaCl_2_, and 1 mL 0.5 g/L thiamine HCl. Micronutrient stock contains 0.18 g/L (NH_4_)_6_Mo_7_O_24_, 1.24 g/L H_3_BO_3_, 0.12 g/L CuSO_4_, 0.8 g/L MnCl_2_, and 0.14 g/L ZnSO_4_.

For production experiments, glycerol stocks of BL21 Star™ (DE3) expression strains containing anthocyanin pathway plasmids and, in some cases, CRISPRi plasmids were first streaked onto LB agar plates containing appropriate antibiotics. Following overnight growth on solid media, cells were inoculated into a single well of a polypropylene deep 48-well plate containing 1 mL liquid media. After 14 h growth in liquid culture at 225 rpm and 37 °C, all strains were subcultured by 50× back-dilution into fresh, room temperature media (20 μL overnight culture into 1 mL AMM), and incubated at 225 rpm and 30 °C. Next, 10 μL 100× substrate (1 g/L (+)-catechin final concentration) stock dissolved in dimethylformamide:ethanol (8:2, v/v) and 10 μL 100× inducer (1 mM IPTG final concentration) stock were added sequentially by multichannel pipette at the optimal cell density as measured on a Biotek Synergy 4 Microplate Reader, and cultures were further incubated at 225 rpm and 30 °C to allow conversion of (+)-catechin to P3G. Cultures were sampled for anthocyanin quantification 18–22 h post-induction with the exception of sampling timecourse studies. All production experiments were performed in biological triplicate, and reported production values represent mean and SEM as quantified by HPLC.

### Anthocyanin quantification by HPLC

**Fig. 6:**
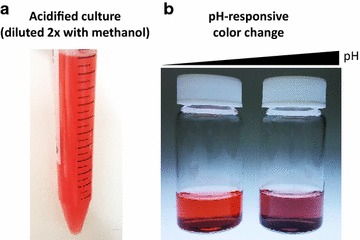
Crude cellular extract containing P3G and C3G exhibits brilliant color associated with anthocyanin production. **a** *E. coli* culture harvested after approximately 12 h induction. 2× dilution of the culture with acidified methanol (1% HCl, v/v) immediately develops the brilliant *red color* shown here, **b** following centrifugation to remove cellular debris, the crude extract exhibits pH-dependent color change. Specifically, C3G and P3G undergo bathochromic shift to longer wavelength (lower frequency) with addition of acid and hypsochromic shift to shorter wavelength (higher frequency) with addition of base, which manifests as the *red* and *purple hues* shown in the vials on the *left* and *right*, respectively

Crude anthocyanin mixtures were extracted from 200 μL culture by addition of an equal volume of acidified methanol (1% HCl, v/v), followed by brief vortexing and centrifugation at 21,000×*g* for 10 min to remove cell debris (Fig. 6). The aglycone cyanidin was not quantified due to rapid degradation in culture, typically resulting in its absence from HPLC traces at final sampling time points. C3G and P3G stock solutions of known concentrations were prepared by dissolving analytical standards (Alkemist Labs) in DMSO and were stored at −20 °C, and standard curves were prepared fresh (by dilution into methanol) at appropriate concentrations for quantifying production from the engineered strains. Extracts and standards were injected (25 μL) for analysis on an Agilent 1200 series HPLC equipped with a ZORBAX SB-C18 StableBond analytical column (150 mm × 4.6 mm, 5 μm) and a diode array detector (DAD). Acetonitrile (solvent A) and water (solvent B), both containing 0.1% formic acid, were used as mobile phases. A flow rate of 1 mL/min was used with the following gradient: 10–40% A (0–10 min) and 10–40% A (10–15 min). C3G (retention time = 4.8 min) and P3G (retention time = 5.5 min) were quantified against standard curves by peak area integration at 520 nm.

## Additional file

**Additional file 1: Table S1.** Synthetic genes used in this study.
