# Supplementary material for: CRISPRi-mediated metabolic engineering of E. coli for O-methylated anthocyanin production
Source: Microb Cell Fact. 2017 Jan 17;16:10. doi: 10.1186/s12934-016-0623-3 (PMC5240198; doi:10.1186/s12934-016-0623-3)
Supplement: Supplementary file 1 — Additional file 1: Table S1. Synthetic genes used in this study. [file 12934_2016_623_MOESM1_ESM.pdf]

SUPPLEMENTARY INFORMATION

CRISPRi-mediated metabolic engineering of *E. coli* for *O*-methylated anthocyanin production

*Brady F. Cress[1], Quentin D. Leitz[1], Daniel C. Kim[1], Teresita D. Amore[2], Jon Y. Suzuki[3], Robert J. Linhardt[1,4,5] and Mattheos A. G. Koffas[1,4]**

[1] Department of Chemical and Biological Engineering, Center for Biotechnology and Interdisciplinary Studies, Rensselaer Polytechnic Institute, 110 8th Street, Troy, NY 12180, USA. [2] Department of Tropical Plant and Soil Sciences, University of Hawaii, 3190 Maile Way, Honolulu, Hawaii 96822, USA. [3] U.S. Department of Agriculture, Agricultural Research Service, Daniel K. Inouye U.S. Pacific Basin Agricultural Research Center, Hilo, Hawaii 96720, USA. [4] Department of Biological Sciences, Center for Biotechnology and Interdisciplinary Studies, Rensselaer Polytechnic Institute, Troy, NY 12180, USA. [5] Department of Chemistry and Chemical Biology, Center for Biotechnology and Interdisciplinary Studies, Rensselaer Polytechnic Institute, Troy, NY 12180, USA.

*Correspondence: RPI, Biotech 4005D, 110 8th Street, Troy, NY, 12180, USA; Tel: +1 518 276 2220; Fax: +1 518 276 3405; Email: koffam@rpi.edu

Table S1 Synthetic genes used in this study

Name	Nucleotide Sequence (5'→3')<br>(*universal conserved flanking sequences*; **start** and **stop** codons; NdeI and XhoI restriction sites)
*Vv*AOMT1_gBlock	*GTTTTCCCAGTCACGACGTTG*CAT**ATG**TCCAGCTCAAGTCATAGGGGCATTCTCAAAACTGAAGCCCTCACAAAGTAT<br>CTATTGGAAACAAGTGCATACCCAAGAGAGCATGAGCAGCTGAAAGGCCTCAGGGAGGCCACGGTAGAAAAGCATAAG<br>TATTGGAGCCTGATGAATGTCCCTGTCGATGAGGGACTGTTCATTTCAATGCTTCTAAAGATCATGAATGCCAAGAAG<br>ACAATAGAGCTCGGAGTGTTCACCGGTTATTCTCTCTTGGCAACAGCTCTTGCCCTGCCTCAAGATGGCAAGATAATA<br>GCGGTTGATCCAGATAAAGAAGCGTACCAAACTGGAGTGCCATTTATCAAGAAGGCAGGTGTGGAGCATAAGATCAAC<br>TTCATTCAATCAGATGCAATGTCAGTTCTAAATGATCTCATTGCCGATGGGAAAGAAGAAGGGACATTGGACTTTGCG<br>ATGGTGGATGCTGATAAGGAGAACTACCTCAACTACCATGAGCTGCTGCTGAAGTTGGTTAGAGTTGGAGGCATAATC<br>GCTTACGATAACACTCTATGGTTTGGTTCAGTAGCGCGATCTGAAGAAGAAGAAATGATGGATTTTGAGAGAGCAGGC<br>AGAGTCCATCTCATGAAGTTGAACAAATTCTTGGCATCAGATCCCAGGGTTGAACTCTCTCACCTTTCCATTGGAGAT<br>GGCGTCGCGCTCTGCAGGCGCCTCTAT**TAG**CTCGAG*CTGTGTGAAATTGTTATCCGCTCA*
*Ckm*OMT2_gBlock	*GTTTTCCCAGTCACGACGTTG*CAT**ATG**GCGGAAAAACCAACATTGATAATACGTCCTAACAAGGGAGTATTACAGAGT<br>GAAGCATTATACGATTATATTGTGGAGACGAGTGTATACCCTCGCGAATCGAAGTTTCTCAAGGAGATTAGGGATGTA<br>ACAGCAAGTCATCCATGGTCTGGTATGTCTAGCAATCCCGATGCAGCTCAGCTGATGGGCATATTATTGAAACTTGCA<br>AATGCGAAGAAAACCATTGAAGTCGGAGTCTTCACCGGATACTCACTTCTACTCACTGCTCTTACCATTCCAAATGAC<br>GGCAAGATCTTAGCCATTGATCCCGATCAAGAGGCGTACGATTTAGGACTGCCTGTTATTAAGAGAGCGGGTGTCGAA<br>CATAAAATCAACTTCGTTGCGGAGGCGGCTCTACCAGTGCTTGACAAGTTGCTAATTGATCCCGAAAATGAAGGGAGT<br>TTCGACTTTGCTTTTGTTGATGCTGACAAAGAAAATTATCTTAATTATCACGAGCGATTACTGAAACTGGTGAAACTT<br>GGTGGGGTAATAGTGTACGACAACACACTGTGGTTCGGTACAGTTGCACTGCCTGAAGACTCTGTAAAAGAGGGCTGG<br>AGACATGGTCGGAAGACAGCATTGGAGGTCAACAAAGCCCTAACATCCGATCCTCGCATCCAACTCGCTCACGTCCCA<br>GTAGGTGATGGGAATGTTATCTGTATTCGCATCCAG**TAA**CTCGAG*CTGTGTGAAATTGTTATCCGCTCA*
*Ph*MF1_gBlock	*GTTTTCCCAGTCACGACGTTG*CAT**ATG**GCAGGCAAAAGCGGACATGGCTCCATTCTTCAAAGTGAAGCCCTCAAGAAG<br>TACATCTTCGAAACTAGTGTGTATCCAAGAGAACACGAGCAACTCAAAGAACTCACACAAGCCTCATTCGATAAGTAT<br>AAAATAGTGAGCTTGATGGGTGTGCCTCCAGATGAAGCCCAATTTCTCTCGATGCTCTTAAAAATAATGAATGCAAAG<br>AAGACAATGGAGATTGGAGTTTTTACCGGTTATTCTCTTCTGGCTACTGCTCTTGCATTGCCAGAAGATGGAAAAATT<br>ATAGCGATTGATCCGGACAGAGAAGCATACGAGGTTGGATTGCCATATATTCAGAAGGCTGGTGTGGAGCATAAGATT<br>GAATTTATTCAATCAGAAGCCTTACCAGTACTCGAAAAACTCCTCTCTAACGGTGAGGAAGAAGGAACATTTGATTTC<br>ATATTCATTGATGCTGATAAGGAGAACTATCTGAAGTACCATGAGATAGTACTAAAATTGGTGAAAGTGGGAGGAGTG<br>ATAGGCTATGACAACACATTATGGTTTGGGACCGTGGCACTTTCAGATGATGATCCTATACCACAAGGCTTAAGAGAA<br>TTGAGGAGATCGGTTTTGAAGATCAACAGTTTTTTAGCTACTGATCCTCGCATTGAATTAGCTCATCTTTCAATTGGT<br>GATGGTCTTACCCTTGGCAGGCGTCTCAGC**TGA**CTCGAG*CTGTGTGAAATTGTTATCCGCTCA*
*Ph*MF2_gBlock	*GTTTTCCCAGTCACGACGTTG*CAT**ATG**GCAGGCAAAAGCGGACATGGCTCCATTCTTCAAAGTGAAGCCCTCAAGAAG<br>TATATCTTTGAAACTAGTGTTTATCCAAGAGAACACGAGCAACTCAAAGAACTCACACAAGCCTCATTCGATAAGTAT<br>AAAATAGTGAGCTTGATGGGTGTGCCTCCAGATGAAGCCCAATTTCTCTCGATGCTCTTAAAAATAATGAATGCAAAG<br>AAGACAATGGAGATTGGAGTTTTTACCGGTTATTCTCTTTTGGCTACTGCTCTTGCATTGCCAGAAGATGGAAAAATT<br>ATAGCGATTGATCCGGACAGAGAAGCATACGAGGTTGGATTGCCATATATTCAGAAGGCTGGAGTGGAGCATAAGATT<br>GAATTTATTCAATCAGAAGCCTTACCAGTACTCCAAAAACTCCTCTCTAACGGTGATGAAGAAGGAACATTCGATTTC<br>ATATTCATTGATGCTGATAAGGAGAACTATCTGAAGTACCATGAGATAGTACTAAAATTGGTGAAAGTTGGAGGAGTG<br>ATAGGCTACGACAACACATTATGGTTTGGGACTGTGGCACTTTCAGATGATGATCCTATACCACAAGGCTTAAGAGAA<br>TTGAGGAGATCTGTTTTGAAGATCAACAGTTTCTTAGCTACTGATTCTCGCATTGAATTAGCTCATCTTTCAATTGGT<br>GATGGTCTAACCCTTGGCAGGCGTCTCAGC**TGA**CTCGAG*CTGTGTGAAATTGTTATCCGCTCA*
*Sl*AOMT_gBlock	*GTTTTCCCAGTCACGACGTTG*CAT**ATG**GCAGAGAAAAGTGGACATGGCTCTATTCTGCGAAGTGAAGCCCTCAAGAAG<br>TATATTTTGGAAACCAGTGTGTATCCAAGAGAGCATGAGCTACTAAAAGAACTCACACAAGCCTCATTCGATAAGTAT<br>AAAATAGTGAGCTTGATGGGTGTGCCTCGCGATGAAGGCCAATTTCTTTCGATGCTTCTAAAGATCATGAATGCCAAG<br>AAGACAATGGAGATTGGAGTTTTCACCGGCTACTCTCTTCTAACTACAGCTCTTGCTTTGCCTGAAGATGGGAAAGTA<br>ATAGCAATTGATCCAGATAAAGATGCATACGAAGTTGGATTGCCATTTATTAAGAAGGCTGGTGTGGAGCATAAGATT<br>CAATTCATTCAATCACAGGCCCTGCCTGTTCTTGAAAAACTCCTCAACGAAAAAGAAGAAGGGACATTTGATTTCATA<br>TTCATTGATGCTGATAAAGAGAACTATTTGAAATACCATGAGATTGTACTAAAATTGGTGAAAGTTGGAGGAGTAGTT<br>GGCTATGATAACACTTTATGGTTTGGGACAGTTGCACTTTCAGAGGATGATCCTATGCCACCAGGATTAAAGGCATTG<br>AGAGGAGTTGTTAGGGAGATCAACACTTTTTTAGCTAATGATCCTAGAATTGAAATGTCTCAACTTTCAATTGGTGAT<br>GGACTTACCCTTTGTCGCCGTCTCTAC**TAG**CTCGAG*CTGTGTGAAATTGTTATCCGCTCA*
